# Profiling of Differentially Expressed Genes in Roots of *Robinia pseudoacacia* during Nodule Development Using Suppressive Subtractive Hybridization

**DOI:** 10.1371/journal.pone.0063930

**Published:** 2013-06-11

**Authors:** Hongyan Chen, Minxia Chou, Xinye Wang, Sisi Liu, Feilong Zhang, Gehong Wei

**Affiliations:** State Key Laboratory of Crop Stress Biology in Arid Areas, College of Life Sciences, Northwest Agriculture & Forestry University, Yangling, Shaanxi, China; University of Connecticut, United States of America

## Abstract

**Background:**

Legume-rhizobium symbiosis is a complex process that is regulated in the host plant cell through gene expression network. Many nodulin genes that are upregulated during different stages of nodulation have been identified in leguminous herbs. However, no nodulin genes in woody legume trees, such as black locust (*Robinia pseudoacacia*), have yet been reported.

**Methodology/Principal findings:**

To identify the nodulin genes involved in *R. pseudoacacia*-*Mesorhizobium amorphae* CCNWGS0123 symbiosis, a suppressive subtractive hybridization approach was applied to reveal profiling of differentially expressed genes and two subtracted cDNA libraries each containing 600 clones were constructed. Then, 114 unigenes were identified from forward SSH library by differential screening and the putative functions of these translational products were classified into 13 categories. With a particular interest in regulatory genes, twenty-one upregulated genes encoding potential regulatory proteins were selected based on the result of reverse transcription-polymerase chain reaction (RT-PCR) analysis. They included nine putative transcription genes, eight putative post-translational regulator genes and four membrane protein genes. The expression patterns of these genes were further analyzed by quantitative RT-PCR at different stages of nodule development.

**Conclusions:**

The data presented here offer the first insights into the molecular foundation underlying *R. pseudoacacia–M. amorphae* symbiosis. A number of regulatory genes screened in the present study revealed a high level of regulatory complexity (transcriptional, post-transcriptional, translational and post-translational) that is likely essential to develop symbiosis. In addition, the possible roles of these genes in black locust nodulation are discussed.

## Introduction

Different leguminous plants interact with appropriate rhizobia genera, leading to the formation of nodules on host plant roots. Rhizobia in root nodules could fix nitrogen to ammonia for plant growth and simultaneously obtain carbohydrates from the plant for sustenance. This perfect, mutually beneficial symbiosis involves a series of complex processes, including rhizobia infection, plant root cell differentiation, nodule formation and nitrogen fixation. Both partners are involved in the interaction by exchanging numerous signal molecules.

Leguminous plants can secrete flavonoids to the rhizosphere in nitrogen-deficient soil. Rhizobia receive signals and subsequently synthesize Nod factors (lipochitooligosaccharides), which are specifically identified by plant receptors. Plant roots commence a series of reactions quickly, including calcium peaking, ion current (Ca^2+^, Cl^−^ and H^+^) formation, root hair deformation, and local cell wall hydrolysis. Then, the rhizobia are wrapped by curled root hairs [1], [2]. Invagination of root hairs leads to the formation of infection threads and meanwhile, nodule primordia form from root cortical cells [3]. When these plant-derived tubular structures enter the root cortex and ramify in the nodule primordium, the rhizobia inside are released into the nodule primordium, leading to the formation of a symbiont [4], [5].

For over two decades, many legume genes, called nodulin genes, which are expressed specially or differently during different stages of nodulation, have been identified using large-scale transcriptome approaches [6]–[9]. Many genes whose transcript or protein levels are modulated during the legume–rhizobium interaction may take part in nodule development. Among these genes, it has been reported that some transcription factors (TFs) and genes involved in signalling play important roles during nodule formation. For example, the *Medicago truncatula* gene *MtHAP2-1* encoding CCAAT-binding TF, its expression is increased during nodule development [10]. The CCAAT-binding TF is regulated by miR169 and uORF1p [11], [12], and plays an important role in nodule development. *Mszpt2-1*, a Kruppel-like TF of the Cys_2_His_2_ (C_2_H_2_) type zinc (Zn) finger family, whose expression is strongly induced in vascular tissues surrounding the symbiotic root nodule, is essential for the differentiation of the nitrogen-fixing zone in alfalfa (*Medicago sativa*) nodules [13]. RabA2, a small GTP-binding protein of the Rab family, the transcriptional activity of which in root hairs is 50% higher than in roots, is required for root hair deformation and the preinfection stages of nodulation in common bean [14].

Although the molecular mechanism of the symbiotic relationship between legumes and rhizobia have been studied intensively in leguminous herbs, such as *M. truncatula* and *Lotus japonicus*, the molecular basis of woody leguminous trees, such as black locust, has yet to be known. The nodulation process of woody plants may be different from that of herbaceous plants. A comprehensive analysis of gene expression profiles during nodulation is critical for understanding rhizobium-legume interactions and subsequent nodule formation. Black locust is a type of quick-growing woody plant with major economic and environmental value. As one of the leguminous plants, the symbiotic nodulation of black locust has caused concern as early as the 1980s. Invasive *R. pseudoacacia* in China or in native America could be nodulated by *Mesorhizobium* and *Sinorhizobium* species with similar nodulation genes [15]. *Mesorhizobium amorphae* CCNWGS0123 is isolated from the root nodule of black locust grown in heavy metal tailings in Gansu Province, China [16]. Plant cross-nodulation test has verified that the strain could infect black locust specifically (data not shown).

To study the molecular mechanism of symbiotic interaction between *R. pseudoacacia* and *M. amorphae* CCNWGS0123 and compare it with those of leguminous herbs, suppressive subtractive hybridization (SSH) was used to separate the nodule-enhanced genes involved in symbiotic nodulaton. PCR-based suppression subtractive hybridization (PCR-SSH) is a simple and powerful technique to identify differentially expressed genes, including genes with relatively low abundance [17]. Profiling of differential expression genes involved in *R. pseudoacacia– M. amorphae* symbiosis were identified in this study.

## Materials and Methods

### Plant materials and rhizobia

*R. pseudoacacia* seeds were surface sterilized in H_2_SO_4_ for 10 min to remove the surface layer wax and then treated with 95% (v/v) ethanol for 1 min, 5% (v/v) NaClO for 10 min, and axenic water eight times. The seeds were placed on 1.2% (wt/vol) agar plates at 22°C for 72 h in the dark for germination. The germinated seeds were then transferred to sterilized glass tubes (30 mm×200 mm) with filter paper close to the inside wall and Fahraeus nitrogen-free nutrient solution [18]. Cotton stoppers were used to seal the tubes. The plant roots were grown in the crack between the filter paper and inside wall of the tubes. The tubes were put into cartons to ensure a dark environment for root growth. The method was used to avoid control roots being infected by other rhizobia in the environment and for easy observation. Cartons with plants were finally put in a growth chamber with 16 h/8 h day/night cycle at 26°C/20°C. After 4 days, the plants were inoculated with *M. amorphae* CCNWGS0123 during the late exponential phase and the same volume axenic water was added to the control roots as mock-inoculation. *M. amorphae* CCNWGS0123 was grown in yeast extract-mannitol medium (YMA) [1.0 g of yeast extract, 5.0 g of mannitol, 0.5 g each of K_2_HPO_4_ and KH_2_PO_4_, 0.2 g of MgSO_4_ ·7H_2_O, 0.12 g of CaC1_2_ ·2H_2_O and 0.1 g of NaCl per liter of distilled water] at 28°C on an orbital shaker (150 rpm) to the late exponential phase.

To observe the infection process of *M. amorphae* CCNWGS0123 clearly, enhanced green fluorescent protein (eGFP) gene was used to mark the bacteria. P*lacZ–egfp* plasmid pMP2444 which derive from the broad-host-range vector pBBR1MCS-5 carrying gentamicin resistance gene was transformed into *M. amorphae* CCNWGS0123 by electroporation as described by Bloomberg [19]. Then, the marked bacterium was named G186. Inoculated plant roots were sampled 0 to 24 days after inoculation (dai). The infection process was observed by fluorescence microscopy.

### Total RNA, mRNA and DNA preparation

For SSH and rapid-amplification of cDNA ends (RACE), total RNA was isolated from inoculated and uninoculated control roots at 1, 4, 6, 8, 11, 14, 18, and 22 d using TRIzol reagent (Takara, Dalian, China) according to the manufacturer's instructions. Samples were treated with DNase I (RNase-free, Takara). The mRNA samples were isolated from total RNA with the PolyATtract^®^ mRNA Isolation System III (Promega, Madison, WI, USA) according to the manufacturer's protocol. For RT-PCR and real-time RT-PCR, total RNA were extracted from the whole-root systems of infected roots at different dai (0, 1, 4, 7, 10, 14 and 18 dai), nodules at 20 dai and uninfected roots at 18 dai (22 d after sowing). Three independent sample sets per stage were collected, weighed, immediately frozen in liquid nitrogen, and then stored at −80°C until extraction of RNA.

### Suppression subtractive hybridization and libraries construction

cDNAs were prepared from 2 μg of poly A^+^ RNA isolated from the total RNA of inoculated and uninoculated roots at 1–22 d. SSH was performed using the PCR-Select Subtractive Hybridization Kit (Clontech, Palo Alto, CA, USA) according to the manufacturer's recommendations. Forward subtracted cDNA library was performed using inoculated root cDNA as tester and uninoculated root cDNA as driver. Reverse subtracted cDNA library was performed using uninoculated root cDNA as tester and inoculated root cDNA as driver. The subtracted PCR products were cloned into pGEM-T vector (Promega) and transformed into *Escherichia coli* JM109 to obtain forward and reverse subtracted libraries. For each library, 600 bacterial clones were selected and grown in Luria-Bertani medium supplemented with ampicillin (100 μg/mL) and glycerol (15%). Two libraries were duplicated and stored at −80°C.

### Differential screening by dot blotting

Inserts of the forward cDNA library clones were amplified by colony PCR using T7 and SP6 promoter primers. Then, 8 µl aliquots of the amplification products were denatured with 5.82 µl of 1 M NaOH (freshly made) and 0.73 µl of 200 mM EDTA (pH 8.2) at 100°C for 10 min and cooled on ice for 10 min. Exactly 1 µl of each mixture was blotted onto a nylon membrane (Hybond^TM^-N^+^; Amersham Pharmacia Biotech Limited, Little Chalfont, Buckinghamshire, UK) and two identical blots were made. Two sets of membranes spotted with the PCR products from the forward SSH cDNA library were prepared for hybridization. The blotted membranes were washed in 2×SSC for 4 min and then baked in an oven at 80°C for 90 min. ^32^P-labelled probes were prepared from the subtracted cDNAs and then hybridized with blots on membranes. One membrane was hybridized with the probe prepared from the forward subtracted cDNAs and the other membrane was hybridized with the probe prepared from the reverse subtracted cDNAs. To produce probes, subtracted cDNAs from infected and uninfected control roots were digested with *Rsa* I to remove the adaptor sequences and were labeled with ^32^P using a Random Primer DNA Labeling Kit (Takara) following the manufacturer's instructions. Then membranes were pre-hybridized at 65°C overnight in pre-hybridization solution [5×SSC, 5×Denhardt's, 0.5% (w/v) SDS and 100 µg/ml salmon sperm DNA]. The pre-hybridization solution was poured off and hybridization solution was added to the hybridized membranes kept overnight at 65°C. After hybridization, the membranes were washed in 2×SSC/0.5% SDS at 65°C for 5 min (twice), in 1×SSC/0.5% SDS for 5 min (twice), in 1×SSC/0.5% SDS for 15 min (once), and in 0.1×SSC/0.5% SDS for 10 min (twice). Signals were visualized by BAS-1800II (Fujifilm, Tokyo, Japan) and analyzed by Multi Gauge Version 3.0 software (Fujifilm).

### Rapid amplification of cDNA ends

RACE was performed using the SMART^TM^ RACE cDNA Amplification Kit (Clontech) according to the manufacturer's instructions. Gene-specific primers were appropriately designed from the known nucleotide sequences (Table S3). The cDNA fragments were amplified using the Advantage 2 PCR Enzyme System (Clontech) and the products were cloned into the T-vector as described above. The full-length cDNAs were assembled by overlapping 5′- and 3′-RACE fragments.

### Sequence analysis and annotation

According to the results of dot-blot hybridization, SSH clones showing a more intense signal (over 5 signal ratios) after hybridization to cDNA probes from infected roots were selected for sequencing. The obtained sequences were analyzed using BioEdit 7.0.9 software (Hall, 1999) to remove those sequenced results of poor quality. The VecScreen system (http://www.ncbi.nlm.nih.gov/VecScreen/VecScreen.html) was used to remove vector sequence contaminations and adaptors. The expressed sequence tag (EST) sequences were grouped into singletons and contigs using TIGR Assembler (http://nbc11.biologie.uni-kl.de/framed/Left/menu/auto/rightigr) and called unigenes. Homology search of these nucleotide sequences were performed in the non-redundant (nr) protein database using the BLASTX program (http://www.ncbi.nlm.nih.gov/BLAST). EST sequences with E value<10^−3^ were classified into putative functional categories according to their putative biological roles and molecular functions. The amino acid sequences of coding regions were deduced from the cDNAs using the ExPASy translate tool (http://web.expasy.org/translate/). InterProScan (http://www.ebi.ac.uk/) [20] and Pfam database (http://www.sanger.ac.uk/Software/) [21] were used to identify conserved amino acid motifs. Multiple alignment of protein sequences were displayed using Clustal W.

### Differential expression validated by RT-PCR and quantitative RT-PCR (qRT-PCR)

To verify the nodule-enhanced expression genes roughly and quickly and also to validate the results from SSH experiments, semi-quantitative RT-PCR was used to verify the differences in the gene expression between infected roots and uninfected control roots at 18 dai. Upregulated genes were chosen for further verification by qRT-PCR. Total RNA was extracted from three independent sample sets per stage and treated with DNase I (RNase-free, Takara). Single-stranded cDNA was synthesized using the PrimeScript^®^ RT Reagent Kit (Perfect Real Time, Takara) and the primers for the chosen genes ware listed in Table 1 and Table S2. SYBR Green real-time PCR assay was performed in a total volume of 20 µl, containing 10 µl of SYBR Green I Master Mix (Takara), 0.8 µl each of the specific primers, and 2 µl of the template cDNA. The amplification program consisted of 1 cycle at 95°C for 30 s, followed by 40 cycles at 95°C for 5 s, and 60°C for 30 s. Melting curves were obtained by slow heating at 0.5°C/s, from 60°C to 90°C, while the fluorescence signal was continuously monitored. Negative control without cDNA template was run with each analysis to evaluate the overall specificity. Amplifications were conducted in 96-well plates in a CFX96 real-time PCR system (Bio-Rad, Hercules CA, USA). All experimental samples were run in duplicate (technical replicate) with three biological replicates for each gene. Analysis of standard error of mean (SEM) was used to determine statistical significance between samples. Quantification of gene expression was performed using the relative quantification (2–^ΔΔC^_T_) method, and data were normalized to 18S rRNA expression. The obtained data was analyzed using the mapping software Origin Pro v8.0 (Origin Lab, Hampton, USA) to create figures.

**Table 1:** List of genes and primers used for real-time RT-PCR.

Accession Numbers	Putative protein function (BLASTX)	E value	Forward primer 5′-3′	Reverse primer 5′-3′
JK974084	C_2_H_2_type zinc finger family protein [*Picea abies*]	4.00E-28	CACTGTAAAACGACGGCACC	TTCTTGAGGCTACCACGGA
JK974195	putative DNA-binding protein [*Glycine max*]	3.00E-27	GGCACCGTCACCAATGTTA	TTGTTGTTCCTGCTGCTGTT
JK974086	*Myb* family transcription factor APL [*Medicago truncatula*]	7.00E-28	TTGCCACATGGTTGCTCA	CCAATGGAAGAAATAGGGGACT
JK974087	MKI67 FHA domain-interacting nucleolar phosphoprotein [*Glycine max*]	3.00E-17	CACAAGAAGTTGGTGGAAAAG	CCGACAATCTCAGGGCAT
JK974089	homeodomain-leucine zipper protein [*Glycine max*]	4.00E-21	TGCTCCTCCATCTTCACATA	TGACAACAGTCCCAAAACAG
JK974090	HAT family dimerization domain containing protein [*Medicago truncatula*]	8.00E-65	GCTCCAAAGTCCAACCCA	TGAAGACCACATCAGGCAGT
JK974091	zinc finger BED domain-containing protein 1-like [*Glycine max*]	4.00E-44	TAATGGACCCTCCCCTGAA	TAGTGTTGCTCCTGACTCTGT
JK974092	transcription factor CCAAT [*Lotus japonicus*]	1.00E-50	GAGGAGGTCTTGACAGTTTTG	CCCCGCACTTGATTCTTT
JK974093	alpha-NAC-related protein [*Glycine max*]	2.00E-14	GGCAGTAGTTCCAAAATCATC	CAGGAGTGTCAAGAAGCAAGG
JK974102	ubiquitin [*Medicago truncatula*]	1.00E-39	TCAGAGCAGTAACGATG TAA	TCTTGTTCTCAGGCTTCG
JK974105	ubiquitin conjugating enzyme [*Cicer arietinum*]	3.00E-25	GCTTTCTATCTGCTCACTGCT	TTACGACACCACCACCTTCC
JK974108	10 kDa chaperonin [*Medicago truncatula*]	7.00E-35	TGGGAAGGTGATAGCAGTTG	CGCAGAAAAGAAAGAAGCA
JK974114	mitochondrial-like, prohibitin-1 [*Glycine max*]	3.00E-29	CGATTATTAGGGCTGAAGGA	TCAAGACCATTCCAAGCAT
JK974116	histone deacetylase [*Medicago truncatula*]	3.00E-74	CGGCAATAAGCGAAGAGT	CATCCAGGTAAGCATCACA
JK974152	proteasome subunit alpha type-1-A-like isoform 1 [*Glycine max*]	5.00E-31	AAGAACTTCATGGGCTCCTC	CCTCCCTCACAATCTCAAAA
JK974137	thioredoxin h [*Ricinus communis*]	4.00E-28	TGGCAACTCCACTTCAATG	TTCCCTTTCTTCACCAACAC
JK974139	methionine sulfoxide reductase [*Morus alba var. multicaulis*]	9.00E-39	AATGTGAAGTGTGAAGCAAG	CTCGTTTAGCCCAAGAATC
JK974129	syntaxin-71-like [*Glycine max*]	1.00E-91	ATCAATGCGGAGATTCGTC	TGTTTTGGCACAGCAGGAG
JK974126	endoplasmic reticulum-type calcium-transporting ATPase [*Medicago truncatula*]	2.00E-134	GCTATCTGGTTATTGGGACG	CAGTTGGCAAGTTGGGAGT
JK974133	RAB1D [*Lotus japonicus*]	6.00E-67	GAATACACCCATTTGGTTAGG	GCTGCTGCCATCAAGAATAG
JK974128	synaptotagmin-1-like isoform 2 [*Glycine max*]	2.00E-96	TGGCTGATGGAGGAAAGG	CCACAACGCCGATAGGAT
JK974158	leghemoglobin [*Sesbania rostrata*]	2.00E-71	ACCTTCCCAACCTCAGTG	CGTCGCTCCATTTGTCTC
AB005552.1	18S rRNA [*Robinia pseudoacacia*]	-	TAGTTGGTGGAGCGATTTGTC	CAGAACATCTAAGGGCATCACAG

### Accession Numbers

The nucleotide sequences of ESTs obtained in this work were registered in dbEST and assigned Genbank accession numbers from JK974084 to JK974197.

## Results

### Stages of G186 infected black locust roots

**Figure 1:**
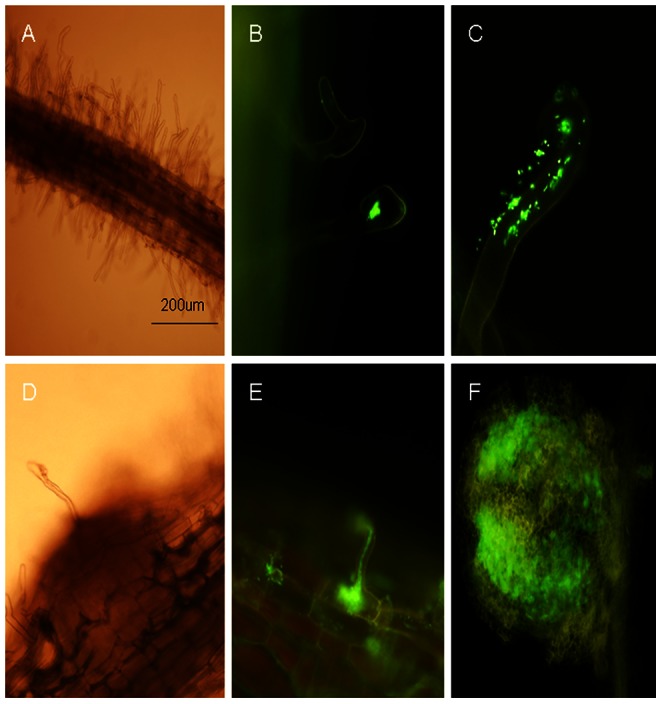
The infection process of G186 into black locust root and formation of nodules were observed by fluorescence microscope. (A) Root hairs deformed at 4 days after inoculation (dai) (White light). (B) G186 was wrapped by curled root hairs at 4 dai. (C) G186 infection of root hairs via infection threads at 7 dai. (D) Formation of nodule primordia at 10 dai (White light). (E) Formation of integral infection thread and release of the rhizobium into the nodule primordium at 10 dai. (F) Fully developed nodule at 18 dai.

Based on preliminary experiments, nodules were detected at approximately 10 dai. It has been shown that nodulation process of black locust is slower than that of *M. truncatula* and *L. japonicus* in which root nodules could be detected at approximately 4 dai [22], [23]. In order to observe the infection process clearly, *M. amorphae* CCNWGS0123 was marked by eGFP gene and designated as G186. Infected roots were periodically sampled to observe the process of G186 infection into locust roots. Under the solution culture condition, nodules were not observed, but extensive root hair cell deformation could be seen and G186 was wrapped by curled root hairs at 4 and 7 dai (Figs. 1A to 1C), indicating strong response of locust roots to G186. At about 10 dai, integral infection threads and nodule primordia emerged. Rhizobia in the infection threads were released into the nodule primordia and this led to the formation of symbionts (Figs. 1D and 1E). Nodules were fully developed at 18 dai (Fig. 1F). The infection of G186 into locust roots was artificially categorized into the following phases: root hair deformation and infection at early stage (0 dai to 7 dai), formation of integral infection threads and nodule primordia at medium-term stage (7 dai to 18 dai) and nodule maturity at late stage (from 18 dai).

### Identification of SSH cDNA sequences

Two subtracted cDNA libraries were constructed by PCR-based SSH to obtain a large amount of ESTs of differentially expressed genes during nodulation in *R. pseudoacacia*. In order to screen nodulin genes involved in nodulation, our studies focused on forward library. Nearly 600 clones were obtained and 371 clones were chosen by colony PCR. The size of the selected clones ranged from 200 bp to 900 bp. Dot blotting was used to remove false positives. The results showed that 97% of the clones were more abundant at the transcript level in the inoculated root cDNA. Then, 200 clones with more than 5 signal ratios after hybridization to cDNA probes from infected roots were finally selected for sequencing and 28 contigs and 86 singletons were obtained by the TIGR assembler. Some unigenes were represented by multiple ESTs, as shown in Table S1, which included contigs with more than two nucleotide sequences.

### Functional annotation and bioinformatics analysis

**Figure 2:**
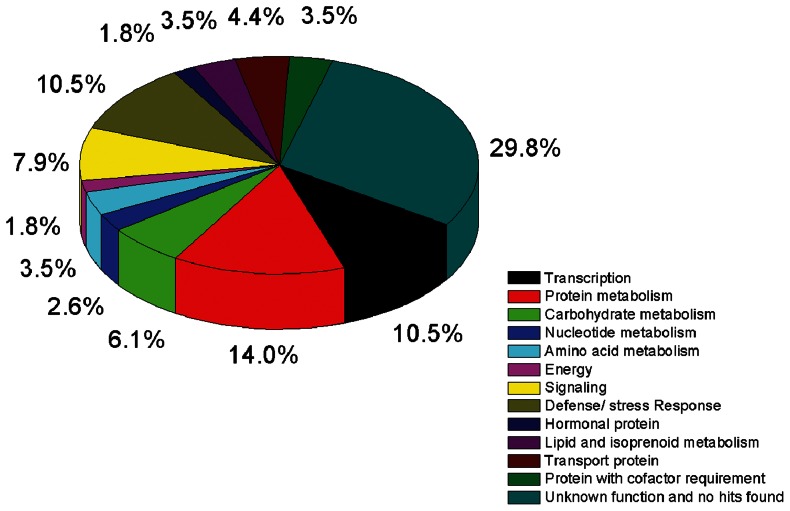
Functional classify of ESTs identified by suppression subtractive hybridization. Unigenes (114 numbers) found to be upregulated in response to nodulation in the present study were grouped into 13 functional categories.

Genome-wide sequencing has not been completed on black locust and gene functional studies on it remain scarce. Thus, using bioinformatics forecasting to obtain the EST functional analysis is particularly important. To assign the putative functions of the cDNAs, the 114 unigenes were searched against the NCBI non-redundant protein database by BLASTX. EST sequences with E value≥10^−3^ were considered as no hit (Table S1). BLASTX annotations of the assembled unigenes demonstrated that 80 (70%) sequences had significant similarities with the sequences in the NCBI nr database, whereas the rest of the unigenes had no homologues at the amino acid level. The ESTs with no hits or no functional annotations had a relatively high abundance (30%), which illustrates the current lack of genomic knowledge on locust. The sequenced ESTs from the forward library were then manually classified into 13 functional categories based on the BLASTX descriptions (Fig. 2). Among the annotated genes, those categories related to Transcription (10.5%), Protein metabolism (14.0%), Defense/stress response (10.5%) and Signaling (7.9%) were overrepresented.

Following their BLASTX annotations, InterProScan annotation tool was used to analyze the functions of these unigenes. Several categories were identified according to cellular component, molecular function, and biological process, then plotted using WEGO [24]. The results showed that the gene products encoded by the collected ESTs might be equally located in extracellular region, intracellular, membrane and organelle (Fig. S1). The main represented categories of molecular functions were: binding, catalytic, electron carrier and transcription regulator. In terms of biological processes, the main ESTs screened from infected root cells were related to cellular process, localization, metabolic process, response to stimulus and transport.

### Confirmation of differentially expressed genes by RT-PCR and qRT-PCR

Regulatory genes are expected to perform crucial functions during nodulation. With a particular interest in these genes, 28 unigenes were preferentially chosen based on the putative annotations linked to transcription, protein metabolism, defense/stress response and signaling. RT-PCR was used to verify the differences in the gene expression between infected roots and uninfected control roots at 18 dai. Primers for RT-PCR of these genes were listed in Table S2. 18S rRNA was amplified as constitutive control and the analyses were repeated at least twice with similar results. Our results revealed that 21 genes were found to be upregulated, while the expression of others 7 genes were not increased in infected roots (data not shown). It could be assumed that the 7 genes were induced either too early or too late after inoculation and this might have attributed to the false-positive results ratio inherent to the SSH technique [25].

**Figure 3:**
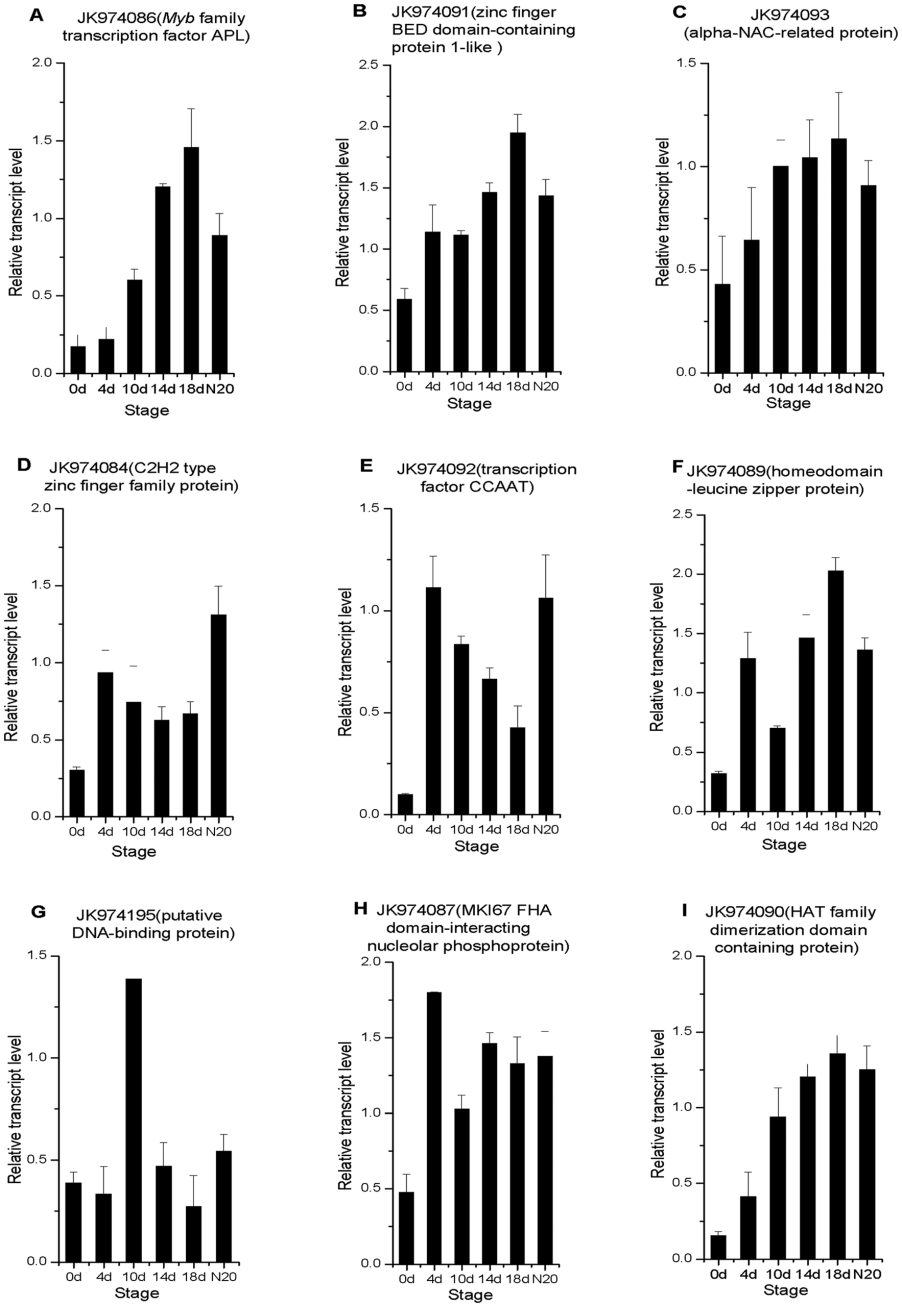
Relative expression levels of nine transcription genes in six developmental stages during nodulation by qRT-PCR. The six stages are: 0 dai, 4 dai, 10 dai, 14 dai, 18 dai, and N20 (nodules removed from 20 dai roots). Data were normalized to 18S rRNA (*R. pseudoacacia*) expression and are presented as mean ± SEM and calculated over biological replicate (n = 2) and technical replicate (n = 3) mRNA.

Among the 21 upregulated genes, nine encoded putative TFs and RNA-binding proteins, four encoded membrane proteins for signaling, and eight encoded post-translational regulators (Table 1). The expression patterns of these genes were accurately quantified by qRT–PCR, using total RNA isolated from the roots at 0, 4, 10, 14 and 18 dai and N20 (nodules removed from 20 dai roots). The results of nine putative transcription genes are shown in Figure. 3. The transcript level of JK974086 encoding a putative *Myb* family transcription factor APL was increased gradually after inoculation and slightly decreased in N20 (Fig. 3A). Similar patterns of expression were seen in JK974091 (zinc finger BED domain-containing protein1-like), JK974093 (alpha-NAC-related protein), JK974089 (homeodomain-leucine zipper protein) and JK974090 (HAT family dimerization domain containing protein) (Figs. 3B, 3C, 3F and 3I). The expression of JK974084 (C_2_H_2_ type zinc finger family protein), JK974092 (transcription factor CCAAT) and JK974087 (MKI67 FHA domain-interacting nucleolar phosphoprotein) showed almost-similar patterns (Figs. 3D, 3E and 3H). The transcriptional levels of the three genes were increased significantly at 4 dai, followed by a decrease, and then tended to increase significantly in N20. JK974195, a putative DNA-binding protein, showed the highest level of transcriptional activity at 10 dai, but levels at the other stages remained relatively unchanged (Fig. 3G).

**Figure 4:**
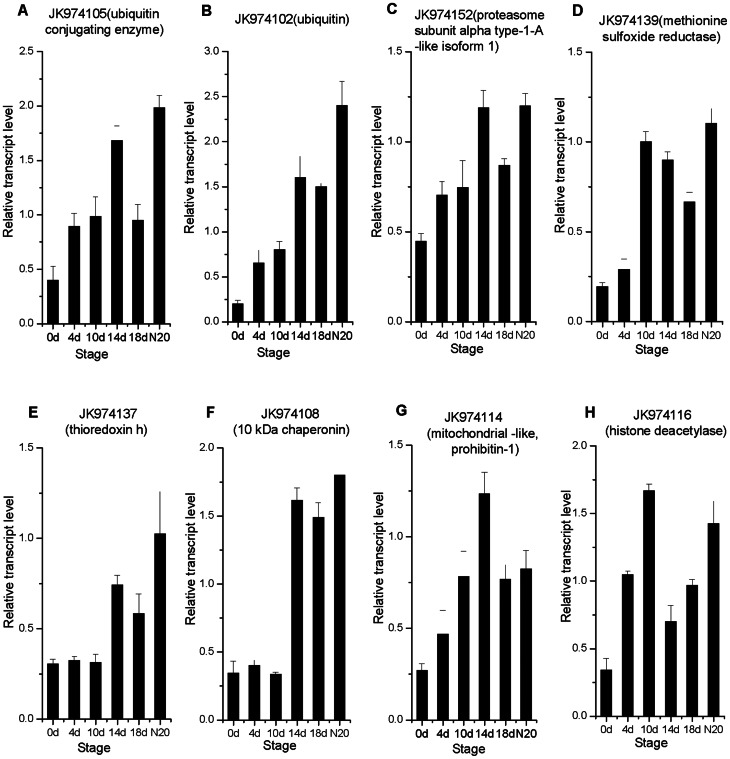
Expression patterns of eight post-translational modification genes in six developmental stages during nodulation by qRT-PCR. The six stages are: 0 dai, 4 dai, 10 dai, 14 dai, 18 dai, and N20. Data were normalized to 18S rRNA (*R. pseudoacacia*) expression and are presented as mean ± SEM and calculated over biological replicate (n = 2) and technical replicate (n = 3) mRNA.

The qRT-PCR results of eight putative post-translational regulator genes are shown in Figure. 4. The expression of JK974105 (ubiquitin conjugating enzyme), JK974102 (ubiquitin) and JK974152 (proteasome subunit alpha type-1-A-like isoform 1), showed similar expression patterns. Their transcriptional activities were enhanced gradually after inoculation and displayed the highest levels in 14 dai root and N20 (Figs. 4A to 4C). On the other hand, JK974137, a putative thioredoxin h and JK974108, a putative 10 kDa chaperonin, demonstrated an increased expression at 14 dai and reached a peak in N20. JK974139, putatively encoding a methionine sulfoxide reductase, was expressed similarly to JK974137 and JK974108 except for an increased expression at 10 dai (Figs. 4E, 4F and 4D). JK974114, encoding a putative mitochondrial-like, prohibitin-1 (Fig. 4G), showed a gradual increase in expression after inoculation and a peak at 14 dai, followed by a decrease in 18 dai root and N20. The transcriptional level of JK974116, a putative histone deacetylase, was significantly different from those of other genes with an improvement in 4, 10 dai roots and N20, and a significant decrease at 14 and 18 dai (Fig. 4H).

**Figure 5:**
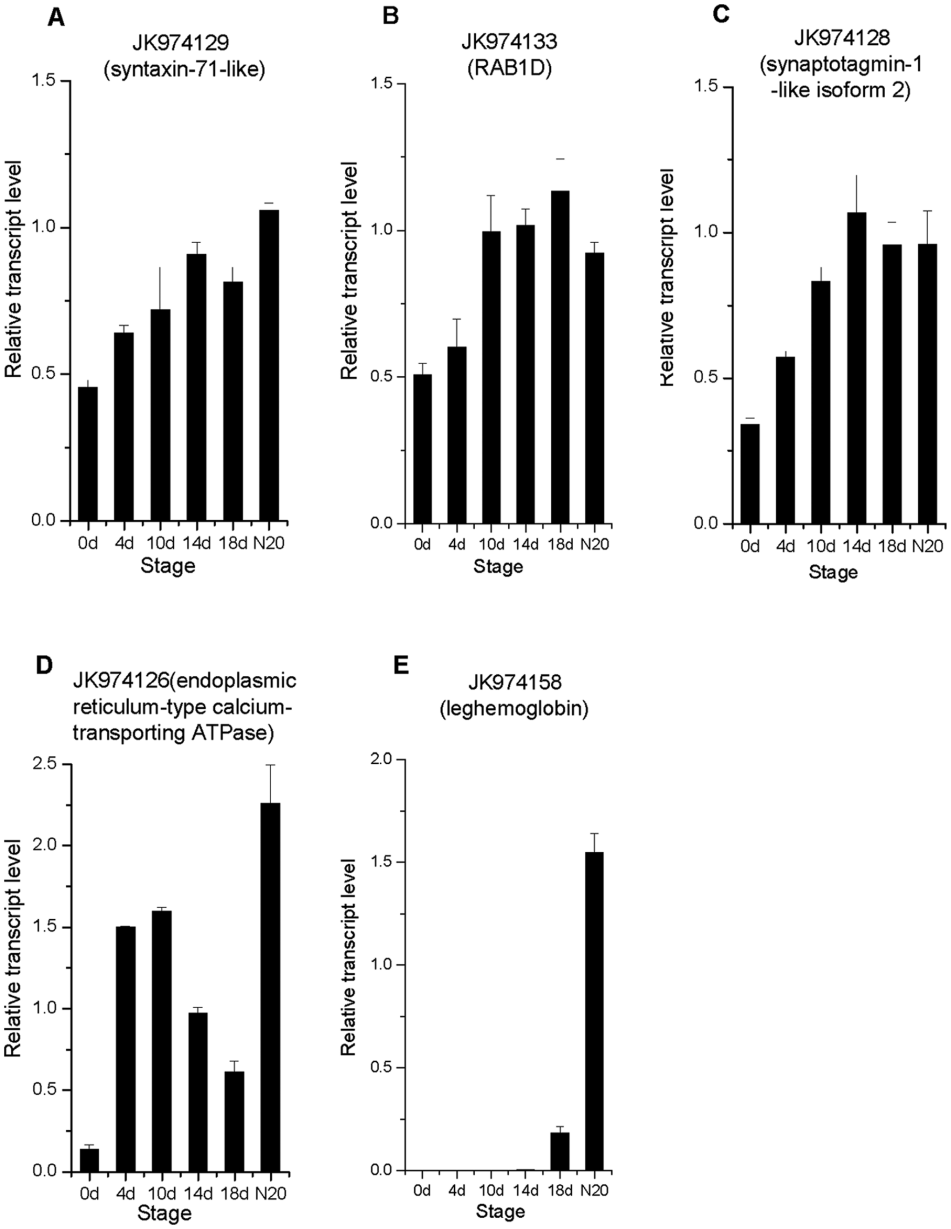
Expression patterns of four membrane protein genes and a leghemoglobin gene. The six stages are: 0 dai, 4 dai, 10 dai, 14 dai, 18 dai, and N20. Data were normalized to 18S rRNA (*R. pseudoacacia*) expression and are presented as mean ± SEM calculated over biological replicate (n  =  2) and technical replicate (n  = 3) mRNA.

Figure 5 shows the transcriptional changes of four putative membrane protein genes: JK974129 (syntaxin-71-like), JK974133 (RAB1D, Rab GTPase family 1), JK974128 (synaptotagmin-1-like isoform 2) and JK974126 (endoplasmic reticulum-type calcium-transporting ATPase). The results indicated that the expression patterns of these genes were increased gradually after inoculation (Figs. 5A to 5C), except JK974126 that resembled JK974116 (histone deacetylase, Fig. 4H) in transcriptional profiles (Fig. 5D).

### Identification of the open reading frames of selected genes by RACE

The full-size cDNA sequences of 8 clones were obtained by RACE (Table S4). Amino acid sequences deduced from these genes showed 59% to 99% homology with the proteins deposited in GenBank databases. Conserved motifs and domains of these genes were predicted by amino acid alignment.

## Discussion

### Identification of nodulin genes in *R. pseudoacacia*

Infection threads appeared in *M. truncatula* roots 24 h after inoculation with *Sinorhizobium* and the rhizobia were released into nodule primordium at 72 h [22]. The nodulation process of locust is slower than that of *M. truncatula*. The infection process of G186 into locust roots could be artificially categorized into early, medium, and late stages (Fig. 1). As a typical late nodulin protein, leghemoglobin always be expressed in mature nodules. The transcription of JK974158 (a putative leghemoglobin) during nodule development could not be detected before 18 dai (Fig. 5E), consistent with our observation that mature nodules could not be found in locust infected roots before 18 dai.

SSH is one of the most powerful methods for identifying differentially expressed transcripts. In the present study, forward and reverse libraries were constructed, which yielded nearly 1,200 positive clones. Dot blot analysis was performed to verify the candidates that differentially expressed in the inoculated roots. About 200 clones were selected from forward library for sequencing, and 114 unigenes were identified as upregulated genes response to symbiosis. Based on the BLASTX annotations, 80 (70%) unigenes were found to encode proteins with either known or putative functions and among these genes, only one sequence JK974144 (a putative auxin-repressed protein) appeared in *R. pseudoacacia* ESTs collected for other purposes in a previous study [26]. So, 113 new genes of *R. pseudoacacia* were identified in the current study. According to the functional annotations, a large proportion of putative gene functions were found to be related to transcriptional regulation, protein synthesis and processing, and signal transduction. So, it is suggested that different levels of regulation, including transcriptional, post-transcriptional, translational and post-translational, may occur during the development of the locust nitrogen-fixing symbiosis.

### Candidate genes involved in transcriptional regulation

Many TF genes have been found to be expressed during nodule development and play important roles in nodulation. Recent studies have indicated that the *Mszpt2-1* gene, encoding a putative C_2_H_2_ type Zn finger protein, is strongly induced in the vascular bundle of roots and nodules. It is an essential gene involved in nitrogen-fixing root nodule organogenesis in *M. sativa* [13]. In the current study, a C_2_H_2_ type Zn finger protein was putatively encoded by JK974084 and expressed at early stage after infection (Fig. 3D). However, JK974084 had only one conserved C_2_H_2_ domain, which was different from *Mszpt2-1* with two C_2_H_2_ domains (Fig. S2A). It is tempting to speculate that different members of this gene family could be induced to exercise specific activities in symbiotic nodulation. Further experiments are needed to sustain the hypothesis. The TF CCAAT (CBF), also known as HAP [10], is a key regulator related to embryogenesis, flowering-time control and abscisic acid responses in various plants [27]–[29]. *MtHAP2-1* is a CCAAT-binding TF from the model legume *M. truncatula* and plays a key role in regulation of nodule meristem. Its expression is regulated by miR169 [11]. JK974092, a putative CCAAT TF, whose transcript was increased significantly at 4 dai (Fig. 3E), had the same conserved region as *MtHAP2-1* (data not shown). It is probably a member of the CCAAT TF family. Therefore, JK974092 might play a role in nodule development in a similar way as the *MtHAP2-1* doe*s*. MKI67 has an RNA recognition motif (RRM) that is a highly abundant domain in eukaryote proteins involved in post-transcriptional gene expression processes. This domain is 90 amino acids long and consists of four-stranded beta-sheet packed against two alpha-helices [30]. Its functional significance remains unclear and is rarely reported in plants. We identified the ORF of a putative MKI67 (JK974087) which contains a consensus sequence of RRM domain (data not shown) and its expression was increased sharply at 4 dai (Fig. 3H). So we speculate that the RRM gene might be induced in plant root cell at the early stage of symbiosis, but its function related to nodulation need further verify. JK974195, a putative DNA-binding protein, belongs to the DUF296 superfamily with unknown function. The AT-hook motif was found in JK974195, which suggested a role in DNA-binding for the protein as a whole [31] (Fig. S2B). The transcript of this gene showed the highest level of activity at 10 dai (Fig. 3G). Although the function of DUF296 in plants was not explored, the relatively increased expression of JK974195 indicated that the member of DUF296 superfamily maybe required for the nodule development. This should be the first report that DUF296 gene is induced during the interaction between leguminous plant and rhizobia.

The others putative TF genes, encoding *Myb* family TF APL (JK974086), Zn finger BED domain-containing protein (JK974091), alpha-NAC-related protein (JK974093), homeodomain-leucine zipper protein (JK974089) and HAT family dimerization domain containing protein (JK974090) showed similar expression patterns, which were increased gradually after inoculation and slightly decreased in N20 (Figs. 3A to 3C, 3F and 3I). In addition, the expression level of JK974089 had a slightly decrease at 10 dai. In soybean, the *Myb* TF gene is involved in nodule formation, named *Control of Nodule Development* (*CND*). When the *CND* gene is silenced, the number of nodules is reduced [32]. Nascent polypeptide-associated complex protein (NAC) exists widely in plants but not in other eukaryotes. Functions of NAC family genes include embryo and shoot meristem development, lateral root formation, auxin signaling, defense and abiotic stress responses [33]. The expression of NAC family genes in senescing leaves have been reported by several groups [34]–[36]. Recent studies have shown that a NAC gene in *M. truncatula* is involved in root abiotic stress responses and symbiotic nodule senescence [37]. The BED Zn finger protein, belonging to a group of resistance (R)-genes, has both a DNA-binding domain and a domain of transposases with unknown function (DUF4413). The BED Zn finger is a novel plant TF that has yet to be reported in terms of its nodulation function [38]. Several plant homeobox genes have possible roles in various stages of vascular tissue differentiation [39], [40]. Soybean homeobox gene *ndx* (nodulin homeobox) is expressed in mature nodules and could also be detected in young nodule primordia [41], [42]. These findings imply that the TF genes identified in our studies are related to black locust nodule development. Here, JK974091 is the first nodule-induced example of BED Zn finger TF involved in symbiotic nodulation of legumes. Therefore, further studies are necessary to define its function.

### Candidate genes involved in post-translational modification

Posttranslational modification (PTM) is one of the later steps in protein biosynthesis and results in the expression of innate functions of proteins. Chaperonins are ‘helper’ molecules required for correct folding and subsequent assembly of some proteins. Many chaperonins are also post-translational regulators. PTM should play a vital role in nodulation because the expressions of several genes involved in ubiquitination, oxidase and chaperonin were increased after inoculation in the present study.

Three genes putatively encoding ubiquitin conjugating enzyme (JK974105), ubiquitin (JK974102), and proteasome subunit alpha type-1-A-like isoform 1 (JK974152), respectively, were upregulated after infection, especially at 14 dai and in N20 (Figs. 4A to 4C). Ubiquitin-conjugating enzyme E2 has an RWD domain, which is identified in WD40 repeat proteins and ring finger domain proteins. CERBERUS, a novel U-box protein containing WD-40 repeats, is strongly induced in developing nodule primordia and the infected zones of mature nodules in *Lotus japonicus*. Mutant experiments have confirmed that this gene plays a critical role in the early steps of infection thread formation as well as in growth and differentiation of nodules [43]. As a chaperone of post-translational modification, ubiquitin has recently been reported to play a role in nodulation [44], [45]. CIP73, containing the Scythe_N ubiquitin-like domain, has been identified to interact with CCaMK, revealing that it is involved in the early signaling way of nodulation [46]. JK974102, belonging to the ubiquitin and the ribosomal L40e family, was induced significantly at the late stage of nodulation in black locust, especially in mature nodules (Fig.4B). So, the role of JK974102 should be different from CIP73, which is a potential regulator involved in nodule formation.

JK974139, JK974137, JK974108 and JK974116 putatively encode methionine sulfoxide reductase, thioredoxin h, 10 kDa chaperonin, and histone deacetylase, respectively. Thioredoxin is a component of a redox system and plays a posttranslational regulatory role in plants. Previous studies have shown that thioredoxin is involved in cell-to-cell communication [47]. In nodule development, a thioredoxin h (*GmTRX*) is induced to reduce reactive oxygen species levels in soybean roots [48]. Methionine sulfoxide reductase (MsrA) is involved in antioxidant defense, protein regulation, and prevention of aging-associated diseases [49]. The function of MsrA in legume symbiosis has not been reported. Chaperonin 10 is an oligomeric molecular chaperone, functioning in protein folding. It is possibly act as an intercellular signal molecule for which could be found on the surface of various prokaryotic and eukaryotic cells [50]. Sequence alignment showed that the predicted JK974108 protein shared highly significant similarity with Cpn10s from various plants (Fig. S2C). Histone deacetylase (HDAC) belongs to zf-UBP superfamily (Zn-finger in ubiquitin-hydrolases and other protein). In the *Arabidopsis thaliana* genome, 16 potential HDAC genes were identified and classified into three families [51]. Functional analysis of HDACs in plants has been studied extensively, yielding evidence that HDACs are involved in the regulation of histone acetylation and thus, gene expression, with consequences for plant morphology and development [52]–[54]. As candidate of posttranslational regulators, the functions of MsrA, Cpn10 and HDAC in legume symbiosis remains unclear. The transcriptional increase of JK974137, JK974108 and JK974116 reflects that these genes are likely to be involved in locust nodulation.

### Candidate genes involved in signaling

Four genes: JK974128, JK974129, JK974133 and JK974126, code for four different member proteins similar to synaptotagmin-1, syntaxin-71, RAB1D and endoplasmic reticulum-type calcium-transporting ATPase, respectively. Synaptotagmin-1 is a Ca^2+^ dependent lipid-binding protein that is required for the maintenance of plasma membrane integrity and cell viability in *Arabidopsis* [55], [56]. The interaction between syntaxin and synaptotagmin is regulated by Ca^2+^ ions [57], [58]. SYP132, a homolog of the syntaxin from *M. truncatula*, which is localized on symbiosome membranes, is involved in infection thread development and the early stages of symbiosome formation [58]. *LjSYP32-1*, a syntaxin-related gene, has been confirmed to contribute to nodule tissue formation in *L. japonicus* [59]. Recent studies have shown that small GTPase is involved in the regulation of nodule-specific trafficking, nodule development and maintenance of nodules in legumes [38]. RabA2, a small GTP-binding protein of the Rab family, is required for root hair deformation and the preinfection stages of common bean symbiosis [14]. Sarco/endoplasmic reticulum-type calcium-transporting ATPase (SERCA) is localized in the nuclear envelope or endoplasmic reticulum. MCA8 is the only one SERCA-type calcium ATPase identified in *M. truncatula* that plays an essential role in the recapture of calcium released into the nucleus during symbiotic calcium signaling [60]. According to previous reports and transcript levels of the four membrane protein genes identified in our study, it is suggested that these candidate of membrane protein genes as signaling molecules might be involved in nodule formation in black locust.

In summary, our study first reports the transcript profiling during *R. pseudoacacia* nodulation. A total of 114 ESTs, whose expression is enhanced in inoculated roots, were successfully separated. Twenty-one upregulated genes were further confirmed by RT-PCR and qRT-PCR. Among these genes, nine genes code for polypeptides showing homology to various TFs and RNA-binding proteins, four membrane protein genes for signaling and eight putative post-translational regulator genes. Further studies using reverse genetics and other tools are recommended to characterize these genes and elucidate their functions in nitrogen-fixing symbiosis.

## Supporting Information

Figure S1 **WEGO classification of the 114 ESTs obtained from the forward SSH library.**
(TIF)

Figure S2 **Clustal W multiple alignments of three amino acid sequences.**
(PDF)

Table S1 **List of cDNA clones of** ***Robinia pseudoacacia*** **identified by suppression subtractive hybridization.**
**(**XLS**)**

Table S2 **List of genes and primers used for RT-PCR.**
(DOC)

Table S3 **Gene-specific primer (GSP) sequences used for RACE.**
(DOC)

Table S4 **List of genes analysed by rapid amplification of cDNA ends.**
(DOC)
