# Supplementary material for: Profiling of Differentially Expressed Genes in Roots of Robinia pseudoacacia during Nodule Development Using Suppressive Subtractive Hybridization
Source: PLoS One. 2013 Jun 11;8(6):e63930. doi: 10.1371/journal.pone.0063930 (PMC3679122; doi:10.1371/journal.pone.0063930)
Supplement: Figure S2 — Clustal W multiple alignments of three amino acid sequences. (PDF) [file pone.0063930.s002.pdf]

Generated by Foxit PDF Creator © Foxit Software
http://www.foxitsoftware.com For evaluation only.

A

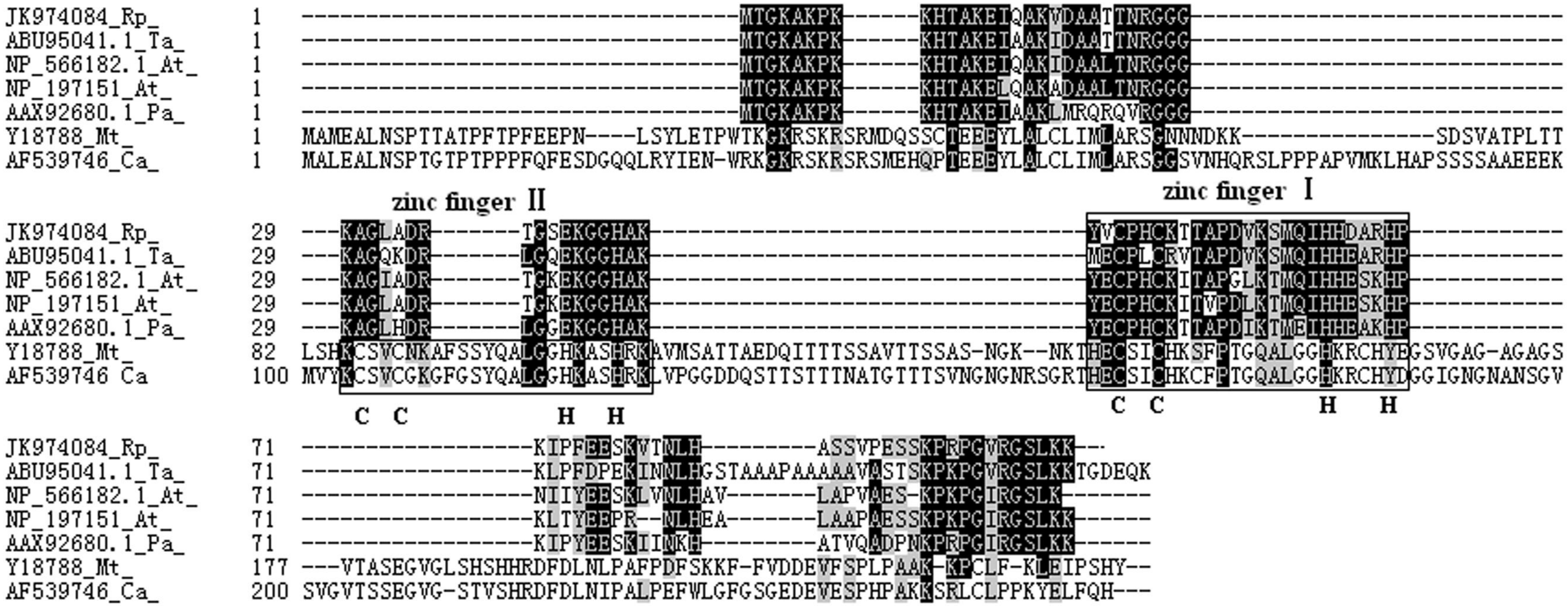

Generated by Foxit PDF Creator © Foxit Software
http://www.foxitsoftware.com For evaluation only.

B

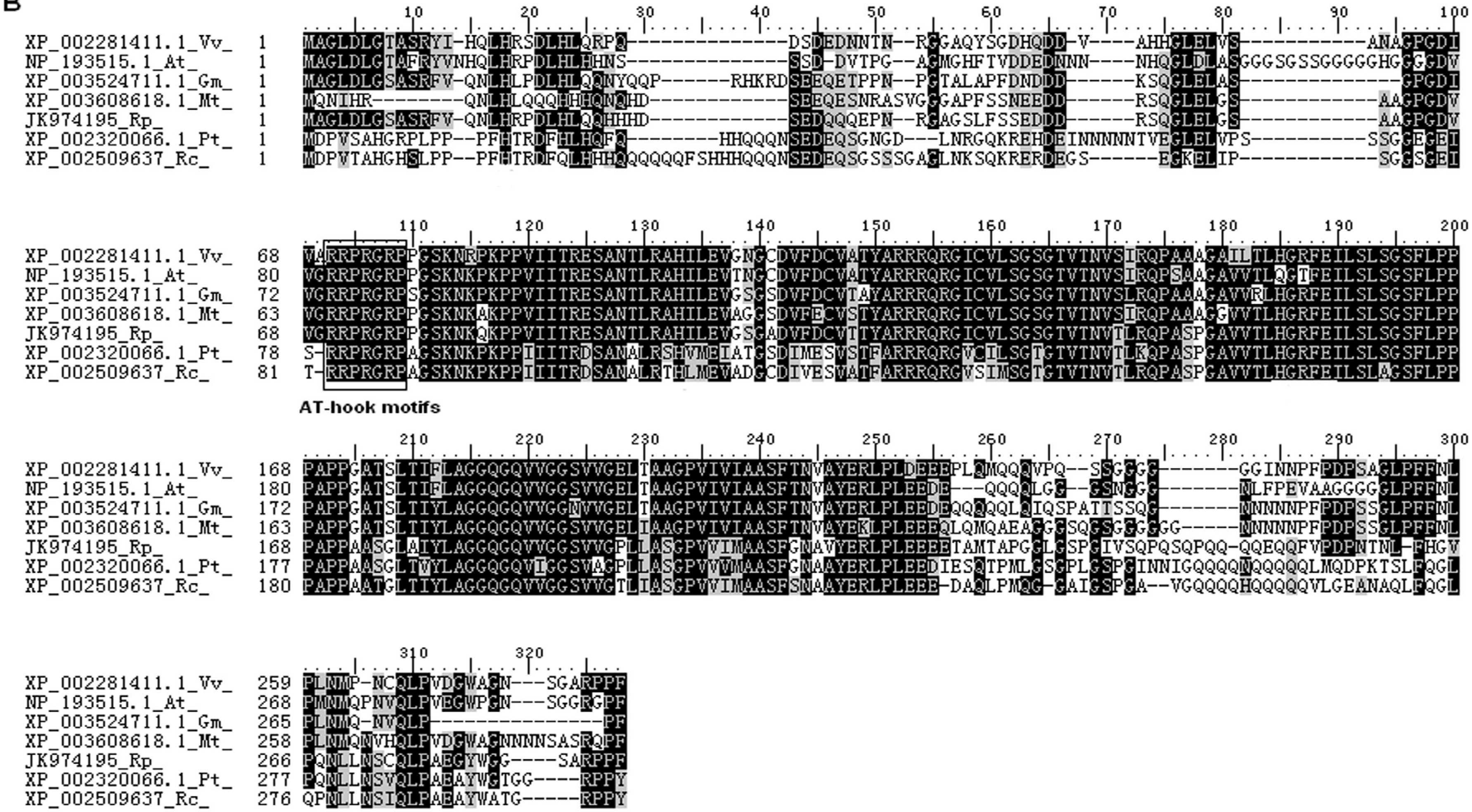

Generated by Foxit PDF Creator © Foxit Software
http://www.foxitsoftware.com For evaluation only.

C

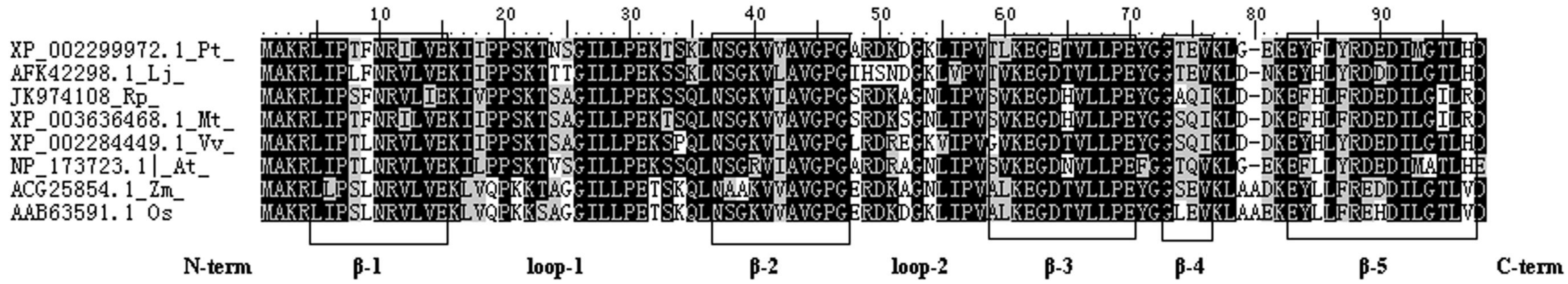

Figure S1 Clustal W multiple alignment of the amino acid sequences. Identical amino acid sequences are highlighted in black and sequence similarities in grey and conserved domains are boxed-in and conserved amino acid residues are marked in the text. (A) Deduced amino acid sequence of predicted C2H2-type zinc finger-containing protein (JK974084-Rp) is compared with other C2H2 type zinc finger plant proteins (sequences of *Arabidopsis thaliana* (NP_566182.1, NP_197151), *Triticum aestivum* (ABU95041.1), *Picea abies* (AAX92680.1), *Medicago truncatula* (EMBL accession no. Y18788) and *Capsicum annuum* (EMBL accession no. AF539746)). C2H2 conserved domains are boxed-in. JK974084 has only one C2H2 domain, which is different from *Medicago truncatula* (Y18788) with two C_2H_2 domains. (B) Multiple alignment of the predicted DNA-binding protein ESCAROLA-like (JK974195-Rp) amino acid sequence with other plant proteins. Sequences of *Glycine max* (XP_003524711.1) , *Vitis vinifera* (XP_002281411.1), *Medicago truncatula* (XP_003608618.1), *Arabidopsis thaliana* (NP_193515.1), *Catharanthus roseus* (ABL63118.1),

Generated by Foxit PDF Creator © Foxit Software
http://www.foxitsoftware.com For evaluation only.

Populus trichocarpa (XP_002320066.1) and *Ricinus communis* (XP_002509637) are compared. Conserved domains of AT-hook motifs are boxed-in, which strongly suggests a DNA-binding function for the proteins as a whole. (C) JK974108-Rp, which encode a 10 kDa chaperonin compared with other plant cpn10 amino acid sequences. Sequences of *Medicago truncatula* (XP_003636468.1), *Vitis vinifera* (XP_002284449.1), *Arabidopsis thaliana* (NP_173723.1), *Populus trichocarpa* (XP_002299972.1), *Zea mays* (ACG25854.1), *Oryza sativa indica* (AAB63591.1) and *Lotus japonicus* (AFK42298.1) are compared. Predicted β strands are boxed-in.
