# Supplementary material for: Profiling of Differentially Expressed Genes in Roots of Robinia pseudoacacia during Nodule Development Using Suppressive Subtractive Hybridization
Source: PLoS One. 2013 Jun 11;8(6):e63930. doi: 10.1371/journal.pone.0063930 (PMC3679122; doi:10.1371/journal.pone.0063930)
Supplement: Table S2 — List of genes and primers used for RT-PCR. (DOC) [file pone.0063930.s004.doc]

**Table S2** List of genes and primers used for RT-PCR.

| **Functional category** | **Accession Number** | **clone ID** | Forward primer 5'-3' | Reverse primer 5'-3' |
| --- | --- | --- | --- | --- |
| Transcription | JK974084 | 1zheng6 | CACTGTAAAACGACGGCACC | TTCTTGAGGCTACCACGGA |
| Transcription | JK974085 | 1zheng7 | AATCCACCGTTTGACCACA | CAGTTTGAGACTAAAGAGGCAGA |
| Transcription | JK974195 | 2zheng98 | GGCACCGTCACCAATGTTA | TTGTTGTTCCTGCTGCTGTT |
| Transcription | JK974086 | 2zheng139 | TTGCCACATGGTTGCTCA | CCAATGGAAGAAATAGGGGACT |
| Transcription | JK974163 | 2zheng279 | CCAATGAACAACTACCTGAA | AACTCCTACCAACTACACAT |
| Transcription | JK974087 | 2zheng168 | CAGAACAGAAGGCTTAGGAA | AGATAGAGGCTGCTGGAAT |
| Transcription | JK974089 | 2zheng219 | GCTCCTCCATCTTCACATACTG | CGGTTCTTCTGATAGCGACTC |
| Transcription | JK974090 | 2zheng240 | CTCTACTTCTTCTGCTCCAA | TCTATCTCAGGACACTTCATC |
| Transcription | JK974091 | 2zheng306 | ATGTCTGTTGAGTGGGTTT | GATGTATTAGGTTGGTGGAAG |
| Transcription | JK974092 | 2zheng325 | AGTCACGCCATCGCCAT | TCACCACCTGCTGCTGTAA |
| Transcription | JK974093 | 2zheng354 | TGATGGCACCAACAATGT | CTGAAGAGGAAGAAGAGGAG |
| Protein metabolism | JK974102 | 2zheng327 | TCAGAGCAGTAACGATGTAA | TCTTGTTCTCAGGCTTCG |
| Protein metabolism | JK974103 | 2zheng341 | AGCATTTTCCCTACTATGTTCTGTG | TTGTTCCTGCTGGACTGTGG |
| Protein metabolism | JK974105 | 2zheng158 | GCTTTCTATCTGCTCACTGCT | TTACGACACCACCACCTTCC |
| Protein metabolism | JK974107 | 2zheng202 | TTCAAGAGGCTGAGAAGTACA | TCTTGGCAATGATAGGGTT |
| Protein metabolism | JK974108 | 2zzheng280 | TGGGAAGGTGATAGCAGTTG | CGCAGAAAAGAAAGAAGCA |
| Protein metabolism | JK974162 | 2z302 | CAAGCACCGTTGAACAAGA | ACATCAGAATGGGACAGCAC |
| Signaling | JK974128 | 2zheng311 | CACAACCACATCCATTCTCTAT | CCAACTACTCAATGACATCCTT |
| Signaling | JK974129 | 2zheng11 | ATCAATGCGGAGATTCGTC | TGTTTTGGCACAGCAGGAG |
| Signaling | JK974126 | 2zheng315 | TCCTCGTCGCAGTGATGAT | GCCACCTCTTCAGTCTCAAG |
| Signaling | JK974133 | 2zheng309 | GAATACACCCATTTGGTTAGG | GCTGCTGCCATCAAGAATAG |
| Signaling | JK974114 | 2zheng336 | CGATTATTAGGGCTGAAGGA | TCAAGACCATTCCAAGCAT |
| Signaling | JK974116 | 1zheng8 | CGGCAATAAGCGAAGAGT | CATCCAGGTAAGCATCACA |
| Signaling | JK974117 | 1zheng15 | GGAAGATTAGATGATGGCAAGG | CACATAAGCAGCCCAAAACA |
| Defense/ stress response | JK974149 | A79 | ACGCTGTCAATAATGGAAGTGCTA | TTGGAAGGAGGGTTTACCTAAGTG |
| Defense/ stress response | JK974134 | 2zheng310 | AAGATTTGTCCGCAGATGG | CCGCAGACTTTTCACCCT |
| Defense/ stress response | JK974137 | 2zheng198 | TGGCAACTCCACTTCAATG | TTCCCTTTCTTCACCAACAC |
| Defense/ stress response | JK974139 | A65 | AATGTGAAGTGTGAAGCAAG | CTCGTTTAGCCCAAGAATC |
