# Supplementary material for: Profiling of Differentially Expressed Genes in Roots of Robinia pseudoacacia during Nodule Development Using Suppressive Subtractive Hybridization
Source: PLoS One. 2013 Jun 11;8(6):e63930. doi: 10.1371/journal.pone.0063930 (PMC3679122; doi:10.1371/journal.pone.0063930)
Supplement: Table S3 — Gene-specific primer (GSP) sequences used for RACE. (DOC) [file pone.0063930.s005.doc]

**Table S3** Gene-speciﬁc primer (GSP) sequences used for RACE

| Accession Number | GSP sequences for 5’ RACE | GSP sequences for 3’RACE |
| --- | --- | --- |
| JK974084 | GCGAGGCTTGGAAGATTCTGGAACACTA | - |
| JK974195 | GACGGTGCCGCTACCACTGAGGA | - |
| JK974087 | TTTGGAGCAGGCTGGATGTTACCG | - |
| JK974090 | TCCGTGATCGTGAGTTTGATGAGGC | - |
| JK974092 | CAGAAAGCGACCACCGCATCCTC | CAGACATGATAGAAGGTGGGCGTGGCA |
| JK974102 | GATGCCACCACGAAGCCTGAGAAC | - |
| JK974105 | CACCTTCCCCTTTTCCCCCATAACA | - |
| JK974108 | CCGCCGTACTCAGGCAACAGCAC | - |
