# Supplementary material for: Profiling of Differentially Expressed Genes in Roots of Robinia pseudoacacia during Nodule Development Using Suppressive Subtractive Hybridization
Source: PLoS One. 2013 Jun 11;8(6):e63930. doi: 10.1371/journal.pone.0063930 (PMC3679122; doi:10.1371/journal.pone.0063930)
Supplement: Table S4 — List of genes analysed by rapid amplification of cDNA ends. (DOC) [file pone.0063930.s006.doc]

**Table S4** List of genes analysed by rapid ampliﬁcation of cDNA ends

| Accession number | Length of polypeptide | Pfam (family) | Description(BLASTP) | Identity(%) |
| --- | --- | --- | --- | --- |
| JK974084 | 104 | Zn_Tnp_IS1 | C2H2-type zinc finger-containing protein [*Arabidopsis thaliana*] | 76% |
| JK974195 | 289 | DUF296 | putative DNA-binding protein ESCAROLA-like [*Glycine max*] | 69% |
| JK974087 | 210 | RRM-1 | MKI67 FHA domain-interacting nucleolar phosphoprotein-like [*Glycine max*] | 83% |
| JK974090 | 176 | no match | HAT family dimerization domain containing protein [*Medicago truncatula*] | 84% |
| JK974092 | 277 | CBF-B/NF-YA | transcription factor CCAAT [*Lotus japonicus*] | 59% |
| JK974102 | 128 | Ubiquitin  Ribosomal protein L40E | ubiquitin [*Medicago truncatula*] | 98% |
| JK974105 | 148 | UQ-con | ubiquitin conjugating enzyme [*Glycine max*] | 99% |
| JK974108 | 97 | Cpn10 | 10 kDa chaperonin [*Medicago truncatula*] | 92% |
